# All-optical manipulation and probing of the d–f exchange interaction in EuTe

**DOI:** 10.1038/srep04368

**Published:** 2014-03-24

**Authors:** R. R. Subkhangulov, A. B. Henriques, P. H. O. Rappl, E. Abramof, Th. Rasing, A. V. Kimel

**Affiliations:** 1 Radboud University Nijmegen, Institute for Molecules and Materials, 6525 AJ, Nijmegen, The Netherlands; 2 Instituto de Fisica, Universidade de Sao Paulo, Caixa Postal 66318, CEP 05315-970 Sao Paulo, Brazil; 3 LAS-INPE, 12227-010 Sao Jose dos Campos, Brazil

## Abstract

We demonstrate that the ultrafast fast dynamics of the d–f exchange interaction, between conduction band electrons and lattice spins in EuTe, can be accessed using an all-optical technique. Our results reveal, in full detail, the time evolution of the d–f exchange interaction induced by a femtosecond laser pulse. Specifically, by monitoring the time resolved dynamics of the reflectivity changes and Kerr rotation of a weak light pulse reflected from the surface of the sample, it is shown that an intense femtosecond light pulse with photon energies higher than that of the bandgap, triggers spin waves in EuTe. The laser-induced spin waves modulate the d–f exchange interaction, and cause the bandgap to oscillate with an amplitude reaching 1 meV, at frequencies up to tens of GHz. The ability to control and monitor the dynamics of the exchange energy with our all-optical technique opens up new opportunities for the manipulation of magnetism at ultrafast time-scales.

Understanding spin dynamics in magnetic materials is a cornerstone for high-speed spintronics and magnetic recording^1^. Femtosecond laser excitation has been shown to trigger a novel type of magnetisation dynamics during which not only spins^2^,^3^, but also the energies of the spin-orbit^4^,^5^ and the exchange interactions^6^,^7^,^8^,^9^,^10^ become time-dependent quantities. Although monitoring of the spin-orbit interaction is possible using X-ray techniques^11^, femtosecond optical probing of the dynamics of the exchange interaction has been an experimental challenge. Here, we exploit the magneto-refractive effect^12^,^13^,^14^ to thoroughly investigate the d–f exchange interaction, between conduction band electrons and lattice spins in the magnetic semiconductor EuTe.

Light will have a direct effect on the Heisenberg exchange interaction, if the Hamiltonian associated with light-matter interaction contains a term: 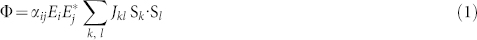
where *E_i_* and 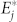 are the *i*- and complex conjugated *j*-components of the electric field of light, **S***_k_* and **S***_l_* are the spins of the *k*th and *l*th ions, *J_kl_* are exchange constants and *α_ij_* is a phenomenological parameter. Equation (1) gives rise to a dielectric permittivity 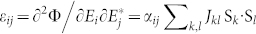, and therefore leads to the magneto-refractive effect^12^,^13^,^14^, i.e., a dependence of the refractive index on the magnetisation of the medium. Equation 1 shows that the dynamics of the magneto-refractive effect reflects time-dependent changes in the the exchange interaction, hence it can be used as a tool to access experimentally the dynamics of the exchange interaction. The Heisenberg antiferromagnet EuTe is an excellent material to test this hypothesis since one can obtain both: (a) a change of the exchange interaction by light^15^,^16^,^17^ (Fig. 1(a)); (b) a strong magneto-refractive effect^13^ (Fig. 1(b)). Indeed, the magnetic properties of EuTe are governed by spins of Eu^2+^ ions (S = 7/2), antiferromagnetically coupled by the superexchange interaction via Te^2+^ (*T_N_* = 9.6 K)^18^,^19^,^20^. Optical properties of the compound are dominated by the electronic transitions from a strongly localised 4*f*^7^ state of the Eu^2+^ ion, to a relatively narrow 5*d*(*t*_2*g*_) conduction band^19^,^21^,^22^ (Fig. 2(a)). An electron in the 4*f*^6^5*d*(*t*_2*g*_) conduction band is characterised by a strong ferromagnetic *d*–*f* exchange interaction^20^,^23^ with Eu^2+^ ions, which is at the origin of the isotropic magneto-refraction. The ferromagnetic *d*–*f* interaction competes with the antiferromagnetic superexchange so that a photo-excitation of electrons from the 4*f*^7^ state into the 5*d*(*t*_2*g*_) band will modify the effective exchange interaction in EuTe, exerting a torque on the sublattices magnetisations (Fig. 1(a)). The *d*–*f* exchange energy operator is given by 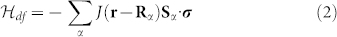
where **r** and **R***_α_* are the radius vectors of the *d*-electron and the *α*th-ion, respectively; ***σ*** is the spin operator for the electron, **S** are spins of Eu^2+^; *J*(**r** − **R***_α_*) are exchange functions. In the electric dipole approximation an optical transition 4*f* → 5*d*(*t*_2*g*_) conserves the spin of an electron, in which case a first order perturbation treatment of 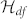 leads to a *d*–*f* exchange energy at *T* = 0 K^24^: 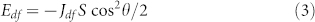
where *J_df_* = 36 meV is an energy exchange constant, S = 7/2, and *θ* is the canting angle between the spins of the two antiferromagnetically coupled sublattices (Fig. 1(a)). Hence the band gap depends on the canting angle *θ*, the equilibrium value of which can be varied continuously by the application of a magnetic field, from *θ* = *π* (at H = 0, when the band gap is maximum) to *θ* = 0 (at *H* > *H_C_* ≈ 8 *T*, when the band gap saturates at a minimum value)^21^,^25^ (Fig. 1(b)). Consequently, an optical excitation of EuTe across the EuTe band gap may trigger dynamics of the ions spins by means of a sudden torque on the lattice spins, produced by the d–f exchange interaction. The optically triggered dynamics of the lattice spins will modulate the bandgap through the d–f exchange interaction, which can be monitored through the magneto-refractive effect.

In order to investigate the laser-induced band-gap dynamics in EuTe we measured the laser induced reflectivity changes using an optical time-resolved pump probe technique (see Methods section and supplementary materials 2). The experiments were performed with a magnetic field applied at 45° to the surface of the sample, which is parallel to the (111) plane (Fig. 2(b)). The central photon energy of the 60 fs probe pulse was chosen at 1.5 eV where the reflectivity is predominantly defined by the energy gap between 4*f*^7^ and 4*f*^6^5*d*(*t*_2*g*_) states. It can be shown that if the energy of the probe photons is lower than the band gap energy, the dynamics of the reflectivity is dominated by the band gap dynamics (see supplementary materials 2). The central photon energy of the 300 fs pump pulse was set to 3 eV and fluence was set to *I* ~ 40 *μJ/cm*^2^ which promotes 10^19^ *cm*^3^. Such photons excite electrons from the 4*f*^7^ band far into the conduction band formed by 5*d* and 6*s* states (Fig. 2(a))^19^. It takes 1–2 ns for these electrons to recombine radiatively back into the 4*f*^7^ state^25^.

It is seen from Fig. 2(c) that at low magnetic fields optical excitation of EuTe leads to a rapid change (within 1 ps) of the reflectivity signal followed by a slow decay on a time-scale of a few 100 ps. In magnetic fields above 3 T oscillations of the reflectivity are clearly resolved. Any photo-induced reflectivity vanishes, when the magnetic field approaches *H_C_* = 8 *T*, when all lattice spin dynamics is quenched. This fact implies that the observed dynamics is due to the dynamics of the energy of the *d*–*f* exchange interaction (see Eq.3). In order to confirm this hypothesis, we focus our discussion on the origin of the oscillations in the reflectivity signal. To this end we also performed time-resolved measurements of the polarisation rotation of the probe pulse upon reflection from EuTe (Fig. 2(d)). In the geometry of the experiment (Fig. 2(b)) the measured signal was proportional to a linear combination of the in-plane and out-of-plane components of the net magnetic moment (see Methods section).

We have performed a Fourier transform of the laser-induced reflectivity and polarisation rotation. The corresponding Fourier spectra are shown in Fig. 3(a) and Fig. 3(b), respectively. It is seen that, in general, the spectra contain two frequencies. The frequencies deduced from the spectra are plotted in Fig. 3(c). To identify these oscillations and link them to the *d*–*f* exchange interaction, we analysed the magnetisation dynamics of the two antiferromagnetically coupled sublattices with magnetisations **M**_1_, **M**_2_ and found that their motion is described by two frequencies of antiferromagnetic resonance (see supplementary materials 4). The low-frequency mode corresponds to the quasi-antiferromagnetic mode (q-AFMR), in which the angle *θ* is modulated while the direction of the net magnetisation vector **M_tot_** = **M**_1_ + **M**_2_ remains nearly constant. In contrast, the opposite behaviour is shown by the high frequency mode, the quasi-ferromagnetic mode (q-FMR), in which **M_tot_** precesses, while *θ* remains nearly constant. Note, however, that for the experimental geometry used in this work, when the magnetic field is applied at 45° to the (111) crystal plane, the separation of normal modes into pure AFMR and pure FMR is only approximate. For instance, the q-AFMR mode is predominantly AFMR, but contains a small admixture of a FMR character, and similarly the q-FMR mode will also hold some AFMR character^27^. Using the formula for the magnetic field dependencies of the spin resonance frequencies (see supplementary materials 4) we fitted the experimental data (see solid lines in Fig. 3(c)). The figure clearly shows that the femtosecond laser excitation is able to trigger both q-AFMR and q-FMR modes in EuTe. As explained at Fig. 1(a), the q-AFMR mode can be excited by means of the laser-induced changes in the exchange interaction. However, this mechanism cannot account for the excitation of the q-FMR mode. We note that contrary to Ref. 28 the oscillations at the q-FMR frequency were observed also when the field was applied in the plane of the sample. It means that a change of the anisotropy caused by a laser-induced heating cannot explain the observed oscillations. We suggest that the q-FMR can be excited by a photo-induced change in the magnetic anisotropy in EuTe. Indeed since the laser excitation brings the Eu^2+^ ions from *f*^7^ (L = 0) to *f*^6^ (*L* ≠ 0) state, this leads to a dramatic enhancement of the spin-orbit interaction and the strength of the magneto-crystalline anisotropy, in particular. Although a femtosecond laser pulse triggers both modes of antiferromagnetic resonance the reflectivity oscillates mainly at the frequency of the q-AFMR mode. As described above, q-AFMR oscillations change the canting angle and thus the energy of the *d*–*f* interaction, whereas for the q-FMR the canting angle remains nearly constant. Hence the observed temporal behaviour of the reflectivity is an excellent demonstration of the fact that time-resolved reflectivity measurements reveal the dynamics of the *d*–*f* exchange energy in the material. At *H* = 5.5 T, the amplitude of the reflectivity oscillations reaches Δ*R*/*R* ~ 0.1% (Fig. 2(c)). Using static reflectivity measurements, the band gap modulation can be estimated to be Δ*E_G_* ~ Δ*E_df_* ~ 1 meV (see supplementary materials 1). This energy corresponds to relatives changes in *d*–*f* exchange energy of 0.01. According to equation (3), such a change in the band-gap corresponds to a canting of the spins up to Δ*θ* ~ 0.5°. One can crosscheck this value using static measurement of the polarisation rotation (supplementary materials 1), which put changes of the sublattices canting angle at Δ*θ* ~ 1° for the quasi-antiferromagnetic mode of precession at *H* = 5.5 T (see supplementary materials 1). The agreement between these two independent estimates represents a clear-cut demonstration of the validity of the model connecting the magneto-refractive effect to the dynamics of the d–f exchange interaction, proposed in this work. To conclude, we demonstrated that the isotropic magneto-refractive effect can be used to manipulate and probe, in full detail, the dynamics of the *d*–*f* exchange interaction in EuTe. This new technique could also be used to investigate the dynamics of the exchange interaction in other materials. Obviously, probing the magneto-refraction originating from *d*–*f* transitions one should be able to examine the dynamics of the exchange energy in other rare-earth compounds. In magnetic materials with superexchange interaction one can probe the strength of the interaction in the spectral range where the magneto-refraction is dominated by charge transfer transitions (Mikhaylovskiy, R. V. et al. Optical manipulation of the exchange spin-spin interaction on a sub-picosecond timescale. Submitted). The magneto-refractive effect in the far-infrared spectral range serves as a measure of magnetoresistance^14^, being thus a time-resolved probe of the strength of the exchange interaction between electrons in the conduction band and localised lattice spins. Our work paves a novel path for investigating time-resolved magnetism of systems in which the exchange interaction is a function of time.

## Methods

The EuTe layer of 4 μm thickness was grown by molecular-beam epitaxy on a (111)-oriented BaF_2_ substrate. The EuTe layer was capped with a 40-nm-thick BaF_2_ protective layer and the high sample quality was confirmed by x-ray analysis. EuTe is a Heisenberg antiferromagnet. The spins of Eu^2+^ are parallel within the (111) *fcc* crystallographic planes and the adjacent planes have alternating spin orientation. The corresponding effective in-plane anisotropy field is about *H_A_* ~ 1 T. Within the (111) plane there is a weak anisotropy *H_a_* ~ 10^−3T^ 26, which aligns the spins along 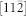 directions. The application of an external magnetic field of about 0.08 T causes a spin-flop transition, followed by a canting of the antiferromagnetically coupled spins.

Experiments were performed in a range of temperatures 1.5–30 K in a gaseous helium atmosphere and magnetic fields of 0–7 T. The magnetic field axis was at 45 degree angle to the sample (111) plane. We employed time-resolved pump-and-probe techniques using a Ti:sapphire laser. The EuTe was excited with an intense 300 ± 50 fs laser (pump) pulse, and we probed with another, much less intense, 60 ± 5 fs laser pulse. Pump fluence density (I) did not exceed I = 100 μJ/cm^2^. By varying the delay between the pump and probe pulses, we were able to study the evolution of the optically induced reflectivity and polarisation changes with subpicosecond temporal resolution. The central photon energy of the probe pulse was chosen at 1.5 eV, while that for the pump was set to 3 eV. The incidence angle of the pump beam was 45 degree to the sample normal, while that of the probe beam was as high as 55 degree. Such geometry permits to measure the dynamics of in-plane and out of plane magnetisation components. In one set of the measurements the rotation of the polarisation plane and the reflectivity changes of the probe beam were measured by means of measuring the differential and sum channel signals of the balanced photo-detector, respectively. Signals were detected with the lockin amplifiers, using the pump repetition rate as the reference frequency and calibrated by the measured DC signal from the balanced detector.

## Author Contributions

R.R.S., A.B.H. and A.V.K. designed the experiment. R.R.S. carried out the experiment and performed the experimental data analysis. P.H.O.R. and E.A. prepared the samples. Th. R. provided the necessary experimental equipment and infrastructure. A.V.K., R.R.S. and A.B.H. wrote the paper with considerable contribution from all the co-authors. A.V.K. supervised the experimental work on the project.

## Supplementary Material

Supplementary Information

## Figures and Tables

**Figure 1:**
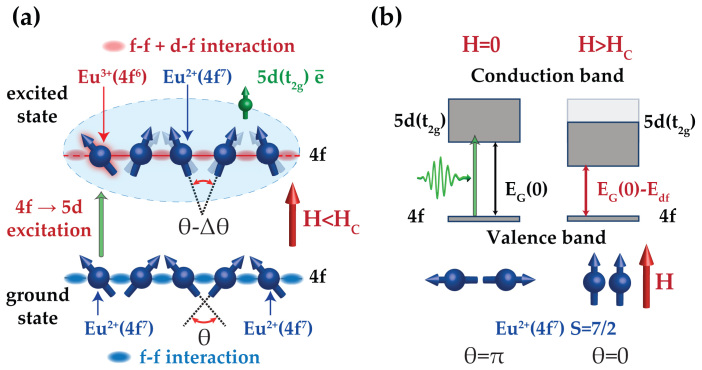
Schematic representation of the interplay between optical and magnetic properties in EuTe. (a) Excitation of an electron from the half-filled 4*f* to the 5*d*(*t*_2*g*_) band of Eu^2+^ induces the *d*–*f* exchange interaction, which competes with the *f*–*f* exchange interaction between Eu^2+^ spins (*S* = 7/2), and causes a canting of the latter; (b) The magnetic field-induced changes of the canting angle between sublattices magnetisation vectors from *θ* = *π* at *H* = 0 to *θ* = 0 at *H* > *H_C_* (in our experiment *H_C_* = 8 *T*). A canting of the Eu^2+^ spins (*S* = 7/2) reduces the *d*–*f* exchange energy and decreases the band-gap.

**Figure 2:**
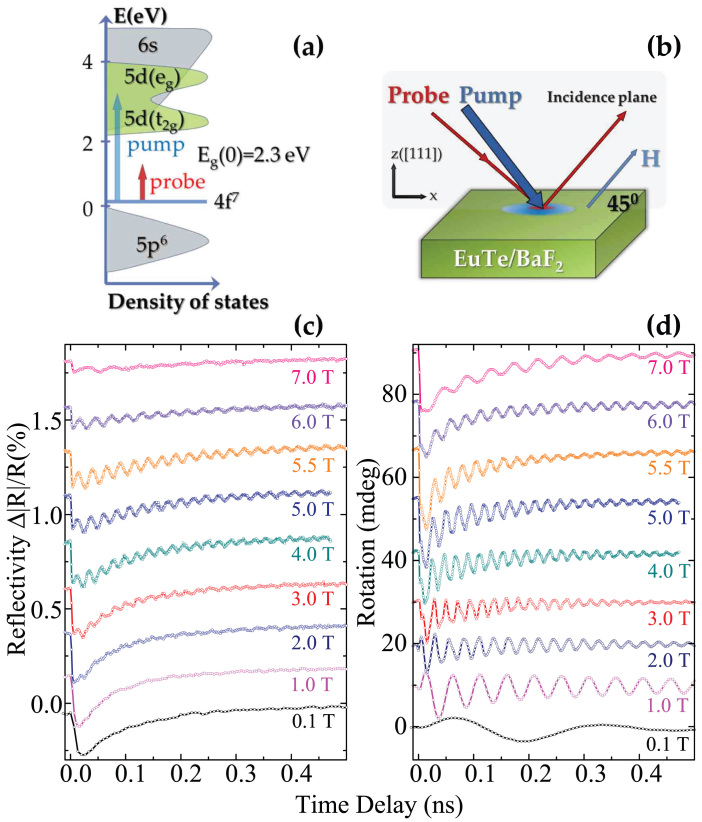
Laser-induced dynamics in EuTe (a) Schematics of the energy bands in EuTe^19^ at *H* = 0 and the photon energies of pump and probe beams. (b) Experimental geometry described in the Method section. (c) Temporal profiles of the reflectivity changes in the sample triggered by a 300 fs pump pulse with fluence density *I* ~ 40 *μJ/cm*^2^ at T = 1.8 K for the range of magnetic fields 0.1–7 T. (d) Temporal profiles of probe polarisation rotation measured at the same conditions as the reflectivity data. The measurements were carried out for a single polarity of the magnetic field.

**Figure 3:**
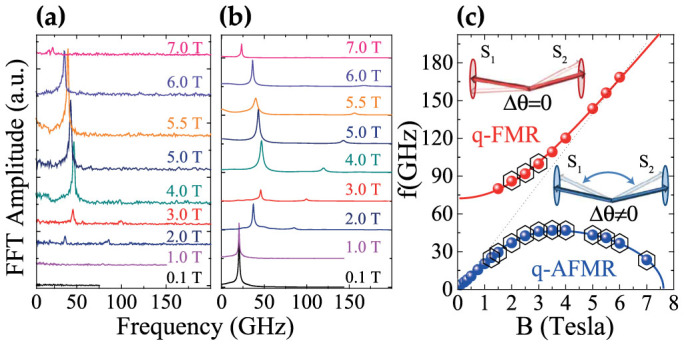
Modes of antiferromagnetic resonance (a) FFT spectra of the reflectivity signal in Fig. 2 (c). (b) FFT spectra of the polarisation rotation signal of Fig. 2 (d). (c) Frequencies of two modes of antiferromagnetic resonance, namely quasi-antiferromagnetic (q-AFMR) and quasi-ferromagnetic (q-FMR). Dots represent the frequency analysis of the experimental data. Hexagons show frequencies extracted from reflectivity data. Lines show the fits deduced from the solution of Landau-Lifshitz equation described in the supplementary materials. The amplitudes of both polarisation rotation and reflectivity oscillations were analysed in the supplementary material 3.
