# Supplementary material for: All-optical manipulation and probing of the d–f exchange interaction in EuTe
Source: Sci Rep. 2014 Mar 24;4:4368. doi: 10.1038/srep04368 (PMC3963036; doi:10.1038/srep04368)
Supplement: Supplementary Information [file srep04368-s1.pdf]

Supplementary materials. All-optical manipulation and probing of the d-f exchange interaction in EuTe

R.R. Subkhangulov[1], A. B. Henriques[2], P. H. O. Rappl[3], E. Abramof[3], Th. Rasing[1] & A. V. Kimel[1]

[1] *Radboud University Nijmegen, Institute for Molecules and Materials, 6525 AJ, Nijmegen, The Netherlands*

[2] *Instituto de Fisica, Universidade de Sao Paulo, Caixa Postal 66318, CEP 05315-970 Sao Paulo, Brazil*

[3] *LAS-INPE, 12227-010 Sao Jose dos Campos, Brazil.*

1. Magneto-optical characterisation of EuTe

To characterise the magneto-optical properties of EuTe we have measured the polarisation rotation and intensity of the light reflected from the sample as a function of an applied magnetic field. The magnetic field vector and the probe beam incidence were at an angle of 45° to the (111) EuTe crystal plane. Figure 1 (a) shows how the angle of polarisation rotation of light depends on the applied magnetic field for two temperatures 1.8 K and 8 K. The external magnetic field above 0.08 T (spin-flop field) changes the canting angle between the magnetisations of the two antiferromagnetically coupled sublattices in EuTe. An increase of the field from 0.08 T to $H_C = 8\ T$ leads to complete alignment of the two sublattices parallel with the field. The critical magnetic field is larger than in Ref. 1 because of the demagnetising factor in the present experimental geometry. An increase of the field up to 6 T leads to a light polarisation rotation of about 1°. Using these values and the formula[2] $\cos\theta = H/H_C$ one can convert the amplitude of the oscillations of the transient polarisation rotation (see Fig. 3 in the main text) into the amplitude of the actual canting angle of the spins of the two antiferromagnetically coupled sublattices ($\theta = \arccos(6/8)$). Our estimate shows that the amplitude of the quasi-antiferromagnetic oscillations corresponds to a canting of about 1°. From the measurements of the reflectivity as a function of the applied magnetic field (see Fig. 1(b)) one can see that an increase of the field from 0 to 6 T leads to a reflectivity change up to 15%. It is known that a complete alignment of the sublattices shrinks the band-gap by 130 meV[1,3]. From this we can estimate that the amplitude of the oscillations of the transient reflectivity shown in Fig. 2 of the main text corresponds to oscillations of the band-gap energy of about 1 meV.

2. Transient reflectivity changes as a probe of band-gap dynamics

Time-resolved reflectivity measurements can serve as a good probe of the band-gap dynamics. Carrier photo-excitation by an ultrashort optical pulse should induce changes in the reflectivity (ΔR/R). ΔR/R is proportional to a sum of changes of the real ($\Delta\varepsilon_r$) and imaginary ($\Delta\varepsilon_i$) parts of the dielectric permittivity:[4]

$$\Delta R/R = \beta_r \Delta\varepsilon_r + \beta_i \Delta\varepsilon_i \tag{1}$$

where β_r and β_i are the Seraphin coefficients. If the energy of the probe pulse is well below the EuTe band gap energy, the absorption coefficient is vanishingly small[5] (less than 0.01 μm^{-1}). In this case we may assume that the probe reflectivity at such an energy is mainly defined by the real part of the dielectric permittivity, i.e. $\Delta R/R \sim \Delta\varepsilon_R$, where $\varepsilon_R = \varphi/(E_G^2 - E^2)$, φ characterises the oscillator strength of the interband transition, and E is the energy of

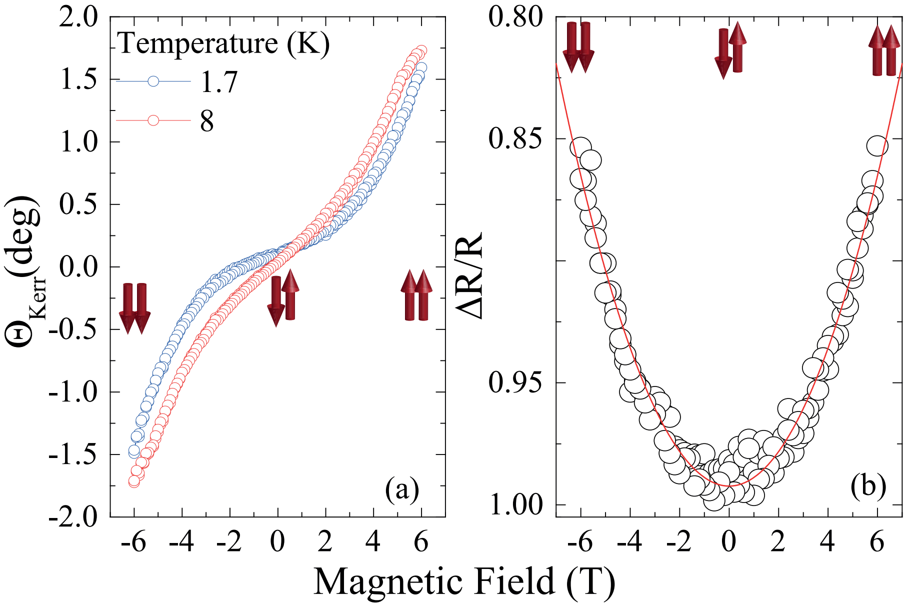

FIG. 1. **Static magneto-optical measurements** (a) Polarisation rotation of the reflected light at the wavelength λ=800 nm as a function of the applied magnetic field measured at two temperatures 1.7 K and 8 K. (b) Reflectivity change in the applied magnetic field measured at 8 K. Arrows demonstrate alignment of the two antiferromagnetic sublattices in the external magnetic field

the probe photon. Consequently, a change of ε_R caused by a small external stimulus can be directly linked to changes of either φ or E_G, respectively:

$$\Delta R/R \sim \Delta\varepsilon_R = \frac{\Delta\varphi}{E_G^2 - E^2} - \frac{2\varphi\, E_G}{\left(E_G^2 - E^2\right)^2}\Delta E_G. \tag{2}$$

Whether the photo-induced reflectivity is defined by changes of φ or E_G can be clarified with the help of measurements of the reflectivity as a function of magnetic field. From Ref. 6 one can conclude that the oscillator strength of the interband transition is insensitive to the magnitude of the magnetic field. Additionally, it is known that application of an external magnetic field effectively reduces the EuTe band-gap due to a reduction of the energy of the $d-f$ exchange interaction[5,7] (see Fig. 1 (b) and Eq. 2 of the main text). Thus field dependent dynamics of the reflectivity must be fully due to the dynamics of the energy of the $d-f$ exchange interaction, ΔE_G, contributing to the band gap energy E_G:

$$\Delta R/R \sim -2\varphi\frac{E_G}{\left(E_G^2 - E^2\right)^2}\Delta E_G. \tag{3}$$

3. Amplitude of oscillation as function of magnetic field

To demonstrate the origin of oscillations observed in our experiment (see Fig. 2 (c),(d) in the main text) we analysed the amplitude of the oscillations in the reflectivity and polarisation rotation measurements. As shown above the reflectivity dynamics is expected to represent the dynamics of the $d-f$ exchange energy. The dynamics of the polarisation rotation is, indeed, that of the magneto-optical Kerr effect, which is determined by the temporal evolution of the net magnetisation. Keeping in mind the spin conservation of electron promoted from the $4f$ to the $5d(t_{2g})$

band, one can write the $d-f$ exchange interaction energy in first order perturbation treatment for $\mathcal{H}_{df}$ defined in Eq. 2 of main text:

$$E_{df} = -\langle\psi_d|\sum_\alpha J(\mathbf{r}-\mathbf{R}_\alpha)\,\mathbf{S}_\alpha\cdot\boldsymbol{\sigma}\,|\psi_d\rangle \tag{4}$$

Here ψ_d is the wave function of a $5d(t_{2g})$ Bloch state. Combining the spatial and the spin part in Eq. 4 and normalising the exchange constant one gets:

$$E_{df} = -J_{df}S\cos^2\frac{\theta}{2} \tag{5}$$

where $S = 7/2$ is the spin of an Eu^{2+} ion, θ is the canting angle between the lattice magnetisations, J_{df} is the energy exchange constant. This expression was obtained assuming that the $5d(t_{2g})$–electron wave function has a uniform distribution over the ions sites. Taking the derivative of (Eq. 5) one obtains the changes of the $d-f$ exchange energy depending on the changes of the canting angle:

$$\Delta E_{df} = J_{df}S\cos\frac{\theta}{2}\sin\frac{\theta}{2}\Delta\theta \tag{6}$$

Combining this expression with[2] $\cos\theta/2 = H/H_C$ one obtains:

$$\Delta E_{df} = J_{df}S\frac{H}{H_C}\sqrt{1-\frac{H^2}{H_C^2}}\Delta\theta \tag{7}$$

This expression represents the changes of the $d-f$ energy as a result of the canting angle changes between the Eu^{2+} ions spins. Therefore the amplitude of the reflectivity oscillations will be given by:

$$A_{\Delta R} \sim \frac{H}{H_C}\sqrt{1-\frac{H^2}{H_C^2}} \tag{8}$$

Polarisation rotation is a measure of the orientation of the net magnetisation. Therefore, the oscillations of the polarisation rotation are due to magnetisation oscillations. The amplitude of the magnetisation oscillations can be derived in the same manner by expressing the net magnetisation as a function of the canting angle $M = 2M_0\cos\theta/2$:

$$\Delta M = -2M_0\sqrt{1-\frac{H^2}{H_C^2}}\Delta\theta \tag{9}$$

where M_0 is the magnetisation of one sublattice. Consequently, the amplitude of the polarisation rotation will be written as:

$$A_{Kerr} \sim 2M_0\sqrt{1-\frac{H^2}{H_C^2}} \tag{10}$$

The equations 8 and 10 show that the field dependencies of the oscillation amplitudes in the reflectivity and the polarisation rotation signal (see Fig. 2(c,d) in the main text) are distinctively different. This was indeed observed in our experiment (see Fig. 2(c,d) in the main text) and shown in Fig. 2 with reasonable agreement.

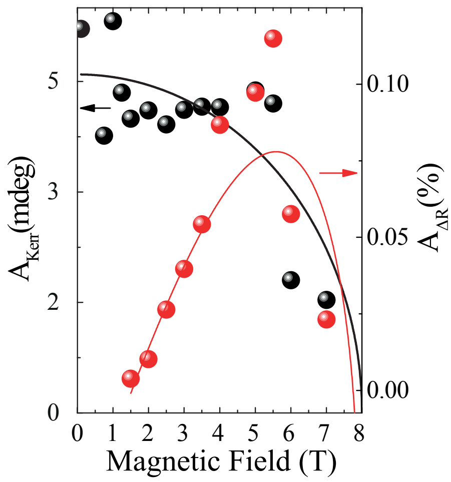

FIG. 2. **Amplitudes of the oscillations in the polarisation rotation and reflectivity measurements.** Dots represent the oscillation amplitudes in polarisation rotation (black dots) and reflectivity (red dots). Lines represent fit using Eq. 8,10 in the present text

.

4. Modes of antiferromagnetic resonance in EuTe

To identify the oscillations observed in the pump probe polarisation rotation measurements and link them to the $d-f$ exchange interaction dynamics, we analysed the magnetisation dynamics of the two antiferromagnetically coupled sublattices in EuTe. To this end we exploited the Landau-Lifshitz equation formalism for antiferromagnets. In the molecular field approximation and in an external magnetic field greater than the spin-flop field $H_{sf} = 0.08\ T$,[8] the total energy of the magnetic system governing the spin arrangement in EuTe is given by[2,9,10]

$$\mathcal{H} = \frac{H_E}{M_0}\mathbf{M}_1 \cdot \mathbf{M}_2 - (\mathbf{M}_1 + \mathbf{M}_2) \cdot \mathbf{H} + \frac{M_0 H_A}{2}\left(\cos\phi_1 - \cos\phi_2\right)^2, \tag{11}$$

where the first, second, and third terms represent the exchange, Zeeman, and out-of-plane anisotropy energy densities, respectively. $H_E = \frac{1}{2}H_C$ is the characteristic $f - f$ exchange field, M_0 represents the magnitude of the sublattice magnetisation, $\mathbf{M}_{1,2}$ are the magnetisation vectors of the sublattices, and $\phi_{1,2}$ are the angles between $\mathbf{M}_{1,2}$, and the axis normal to a (111) plane. Equation Eq. 11 leads to an effective magnetic field of

$$\mathbf{H}_1 = -\nabla\mathbf{M}_1\mathcal{H} = -\frac{H_E}{M_0}\mathbf{M}_2 + \mathbf{H} - \hat{z}\frac{H_A}{M_0}\left(M_{1z} - M_{2z}\right) \tag{12}$$

acting on the first sublattice, where $\hat{z}$ is a unit vector along the axis normal to a (111) plane. If in Eq. 12 we exchange the subindexes $1 \leftrightarrow 2$, the field acting on the second sublattice is obtained. The effective magnetic fields, $\mathbf{H}_1$ and $\mathbf{H}_2$, can be plugged into the Landau-Lifshitz equations, to determine the time-evolution of the staggered oscillating magnetisation vectors. By solving the Landau-Lifshitz equation, we find that the periodic motion of $\mathbf{M}_1$ and $\mathbf{M}_2$ is described by two normal frequencies:

$$\omega_{\pm}^2 = \frac{\gamma^2}{2}\left[\left(1 - \frac{H_A}{H_E}\right)H^2 + 4H_A H_E \pm \sqrt{\left[\left(1 + \frac{H_A}{H_E}\right)H^2 - 4H_A H_E\right]^2 + 2\frac{H_A}{H_E}\left(4H_E^2 - H^2\right)H^2}\right]. \tag{13}$$

where $\gamma = g_J e/2m$, and $g_J = 2$ for Eu$\left({}^8S_{7/2}\right)$. For the Voigt geometry when the magnetic field is in plane there are two pure oscillation modes: quasi-ferromagnetic (q-FMR) mode and quasi-antiferromagnetic (q-AFMR) mode. In the former mode the net magnetisation vector $\mathbf{M}_{tot} = \mathbf{M}_1 + \mathbf{M}_2$ precesses around the applied field, but the angle θ and the magnitude of the net magnetisation remains constant. In the latter the angle θ and the net magnetisation $\mathbf{M}_{tot}$ oscillates, whereas the direction of the $\mathbf{M}_{tot}$ remains nearly constant. In the present experimental geometry (angle between magnetic field vector and [111] is 45 degree) these two modes are mixed: both the direction of the net magnetisation and the canting angle are changed during precession. Above description of q-FMR and q-AFMR is only applicable in limiting cases of the external magnetic fields $H < H_E$ or/and $H > H_E$.

The equation 13 was used to fit the frequencies detected in the laser induced dynamics as a function of magnetic field (see Fig. 3 in the main text). Using two parameters in the fit setting H_E and H_A, we found an excellent agreement for $H_A = 0.7$ T and $H_E = 3.9$ T. Those values are close to those measured in bulk EuTe.[1,11] This clearly shows that the oscillations observed in the dynamics of the magneto-optical signal are the two modes of spin resonances in the antiferromagnet.

[1] Heiss, W. et al. Magnetic polaron induced near-band-gap luminescence in epitaxial EuTe. *Phys. Rev. B* **70**, 035209– (2004).
[2] Henriques, A. B. et al. Magnetic field dependence of the circular dichroism in EuTe. *J. Phys.: Condens. Matter* **19**, 406234 (2007).
[3] Henriques, A. B. et al. Zero-phonon emission and magnetic polaron parameters in EuTe. *Appl. Phys. Lett.* 091906 (2011).
[4] Ju, P. & Cardona, M. Fundamentals of Semiconductors: Physics and Materials Properties, chap. 6, 307 (Springer, Berlin, 1999).
[5] Henriques, A. B. et al. Band-edge polarized optical absorption in europium chalcogenides. *Phys. Rev. B* **72**, 155337 (2005).
[6] Henriques, A. B. et al. Modeling the dichroic absorption band edge and light-induced magnetism in EuTe. *Phys. Rev. B* **77**, 035204 (2008).
[7] Wachter, P. The optical electrical and magnetic properties of the europium chalcogenides and the rare earth pnictides, vol. 3, 189 (Taylor & Francis, 1972).
[8] Battles, J. W. & Everett, G. E. Antiferromagnetic-resonance measurements in EuropiumTelluride. *Phys.Rev. B* **1**, 3021 (1970).
[9] Turov, E. A. Physical Properties of Magnetically Ordered Crystals (Elsevier Science & Technology Books, 1965).
[10] Gurevich, A. G. & Melkov, G. A. Magnetization Oscillations and Waves (CRC Press, 1996).
[11] Demokritov, S. O., Kreines, N. M. & Kudinov, V. I. Inelastic scattering of light in the antiferromagnet EuTe. *Sov. Phys. JETP* **65** (1987).
